# Supplementary material for: Comparative multiomics analysis of cell physiological state after culture in a basket bioreactor
Source: Sci Rep. 2022 Nov 23;12:20161. doi: 10.1038/s41598-022-24687-4 (PMC9686226; doi:10.1038/s41598-022-24687-4)
Supplement: Supplementary file 1 — Supplementary Information 1. [file 41598_2022_24687_MOESM1_ESM.zip › raw data/Proteomic raw data/6.KEGGenrichment/EG.vs.CG/ALL/pathway/src/04933.html]

map04933


- K06527

- Down regulated proteins

A0A0D9S6M6(Description:Uncharacterized protein;Gene:VCAM1;log2FC:-1.9990)

- K06236, K19720, K06237

- Down regulated proteins

A0A0D9R486(Description:Collagen type IV alpha 3 chain;Gene:COL4A3;log2FC:-1.4852)

A0A0D9RY11(Description:Collagen type IV alpha 2 chain;Gene:COL4A2;log2FC:-1.7446)

A0A0D9RY12(Description:Collagen IV NC1 domain-containing protein;Gene:COL4A1;log2FC:-1.2021)

- K05717

- Down regulated proteins

A0A0D9R9X4(Description:Fibronectin;Gene:FN1;log2FC:-1.1754)

Close
